# Interferon gamma inhibits the differentiation of mouse adult liver and bone marrow hematopoietic stem cells by inhibiting the activation of notch signaling

**DOI:** 10.1186/s13287-019-1311-0

**Published:** 2019-07-16

**Authors:** Yuhong Qin, Keke Fang, Nan Lu, Yuan Hu, Zhigang Tian, Cai Zhang

**Affiliations:** 1 Institute of Immunopharmacology and Immunotherapy, School of Pharmaceutical Sciences, Shandong University, Jinan, 250012 Shandong China; 2 Institute of Diagnostics, School of Medicine, Shandong University, Jinan, 250012 Shandong China; 3 Institute of Immunology, School of Life Sciences, University of Science and Technology of China, Hefei, 230026 Anhui China

**Keywords:** Liver, Hematopoietic stem and progenitor cell, Differentiation, γδT cells, Notch signaling, JAK/STAT

## Abstract

**Background:**

The paradigm of hematopoietic stem and progenitor cells (HSPCs) has become accepted ever since the discovery of adult mouse liver hematopoietic stem cells and their multipotent characteristics that give rise to all blood cells. However, differences between bone marrow (BM) and liver hematopoietic stem cells and the hematopoietic microenvironment remain poorly understood. In addition, the regulation of the liver hematopoietic system remains unknown.

**Methods:**

Clone formation assays were used to confirm that the proliferation of adult mouse liver and bone marrow HSPCs. Model mice with different interferon gamma (IFN-γ) levels and a co-culture system were used to detect the differentiation of liver HSPCs. The γ-secretase inhibitor (GSI) and the JAK/STAT inhibitor ruxolitinib and cell culture assays were used to explore the molecular mechanism by which IFN-γ impairs HSPC proliferation and differentiation.

**Results:**

The colony-forming activity of liver and bone marrow HSPCs was inhibited by IFN-γ. Model mice with different IFN-γ levels showed that the differentiation of liver HSPCs was impaired by IFN-γ. Using a co-culture system comprising liver HSPCs, we found that IFN-γ inhibited the development of liver hematopoietic stem cells into γδT cells. We then demonstrated that IFN-γ might impair liver HSPC differentiation by inhibiting the activation of the notch signaling via the JAK/STAT signaling pathway.

**Conclusions:**

IFN-γ inhibited the proliferation of liver-derived HSPCs. IFN-γ also impaired the differentiation of long-term hematopoietic stem cells (LT-HSCs) into short-term hematopoietic stem cells (ST-HSCs) and multipotent progenitors (MPPs) and the process from LSK (Lineage^−^Sca-1^+^c-Kit^+^) cells to γδT cells. Importantly, we proposed that IFN-γ might inhibit the activation of notch signaling through the JAK/STAT signaling pathway and thus impair the differentiation process of mouse adult liver and BM hematopoietic stem cells.

**Electronic supplementary material:**

The online version of this article (10.1186/s13287-019-1311-0) contains supplementary material, which is available to authorized users.

## Background

Hematopoietic stem cells (HSCs) are the most effective cells in the process of hematopoiesis. HSCs can self-renew and can generate all blood cell lineages via step-wise differentiation into downstream progenitors and fully mature cells [1]. In mammals, the first blood cells emerge within the yolk sac before circulation is initiated [2]. Later in gestation, the fetal liver becomes the main organ harboring hematopoietic activity [3]. After birth, bone marrow is the most important hematopoietic organ, which can replenish all blood cell types throughout life [4–7]. However, some studies have shown that a small number of HSCs persist in the adult liver [8–12]. Transplantation experiments have shown that the HSCs in the liver retain their hematopoietic capacity and can differentiate into various mature immune cells [10, 13–15]. To some extent, these liver HSCs can provide compensatory hematopoiesis when bone marrow (BM) function is restrained [10]. The differences between BM and liver HSCs and the hematopoietic microenvironment are worthy of research attention.

Under normal circumstances, the proliferation, self-renewal, and differentiation of HSCs occur in a relatively balanced state. However, under some conditions, such as infection and inflammation, the hematopoietic microenvironment of the BM may be dramatically altered and this equilibrium may be disrupted [16]. Currently, increasing research is focusing on the regulation of the hematopoietic system. The hematopoietic system can be influenced by proinflammatory cytokines, such as interleukin, tumor necrosis factor α, granulocyte colony-stimulating factor, and interferon-α [17]. Among these cytokines, the role of interferon-gamma (IFN-γ) on hematopoiesis has received increased attention. IFN-γ impairs the formation of several hematopoietic lineages, such as B cells [18], erythrocytes [19], and eosinophilic [20] and neutrophilic granulocytes [21]. Some studies reported that increased IFN-γ production has been associated with BM failure in patients with aplastic anemia and chronic myeloid leukemia [22, 23]. In fact, the development of marrow failure can be ameliorated using broadly acting immunosuppressive agents and monoclonal antibodies specific for IFN-γ [24, 25]. In addition, IFN-γ neutralization improves the in vitro capacity of hematopoietic progenitors from patients with aplastic anemia [26]. Thus, IFN-γ has indispensable effects [27, 28] in BM hematopoiesis and has important clinical significance [21, 27, 29, 30]. IFN-γ is overproduced in patients with aplastic anemia (AA) and markedly affects the clinical outcome and patient survival [31]. However, whether IFN-γ has any effects on adult liver hematopoiesis remains poorly understood.

Therefore, the present study aimed to examine the function of IFN-γ on hematopoietic stem and progenitor cells (HSPCs) in the adult mouse liver. The results showed that IFN-γ inhibited the proliferation ability, and the differentiation of adult liver HSPCs, including the differentiation of HSPCs into common lymphoid progenitor cells (CLP) and the LSKs into γδT cells. We also provided a molecular mechanism by which IFN-γ impairs HSPC proliferation and differentiation. We believe that these findings will help to explain the effect of IFN-γ on hematopoiesis in chronic inflammatory diseases and provide a better understanding of the underlying mechanism of hematological malignancies.

## Methods

### Mice and IFN-γ treatment

Six- to 8-week-old male C57BL/6j mice were obtained from Beijing Hua Fukang Bioscience Co. Ltd. (Beijing, China). IFN-γ−/− mice in a C57BL/6 J background were obtained from The Jackson Laboratory (Bar Harbor, ME, USA). The IFN-γ−/− mice appeared normal, with a healthy status, and were viable and fertile in a clean environment; however, they displayed reduced macrophage function in response to pathogens, as characterized by The Jackson Laboratory [29, 32].

Plasmid pLive-IFN-γ, which stably expresses cytokine IFN-γ and encodes kanamycin resistance, was donated by the College of Life Sciences, University of Science and Technology of China. Plasmid pLive-IFN-γ plasmid was injected into 6- to 8-week-old C57BL/6j mice at 5 μg per mouse in 2 mL of 0.9% sodium chloride via hydrodynamic injection. The control mice were injected with empty pLive plasmid. All mice were kept under pathogen-free conditions according to the guidelines of the Institutional Animal Care and Use Committee at Shandong University.

### Colony-forming unit assay

A total of 2 × 10^5^ bone marrow or liver mononuclear cells (MNCs) or 5000 sorted LSK (Lineage^−^Sca-1^+^c-Kit^+^) cells were suspended in 2 mL of methyl cellulose semisolid medium (1.5% methylcellulose in Iscove’s modified Dulbecco’s medium (IMDM)) and seeded into a 24-well culture plate in which stem cell factor (SCF) 50 ng/mL, Fms-related tyrosine kinase 3 (FLT3) 50 ng/mL, interleukin (IL)-3 20 ng/mL, IL-7 20 ng/mL, macrophage colony stimulating factor (M-CSF) 50 ng/mL, and granulocyte-macrophage colony-stimulating factor (GM-CSF) 50 ng/mL were added. If IFN-γ was needed, it was added at 20 ng/mL. SCF, FLT3-L, IL-3, IL-7, and IFN-γ were purchased from PeproTech (Rocky Hill, NJ, USA). The cells were incubated at 37 °C in 5% CO_2_ for 14 days. The number of colonies (with > 50 cells) of colony-forming unit-granulo-macrophage (CFU-GM) and CFU-macrophage (CFU-M) was counted under a light microscope on day 14.

### Cell separation, cell staining, and flow cytometry

Bone marrow and liver MNCs were harvested from the mice. Single-cell suspensions were blocked with an Fc receptor CD16/CD32 (0.5 μL/10^6^ cells) at room temperature. After 10 min, the cells were stained with a cocktail of antibodies. After 30 min of staining at room temperature, the cells were washed with a 1 × phosphate buffer solution.

Monoclonal antibodies for murine lineage antibodies cocktail comprised of Peridinin chlorophyll protein complex (PerCP)-cy5.5 (clone, 51-9006977), CD127-Phycoerythrin (PE)-Cy7 (clone, A7R34), marker of proliferation Ki-67 (Ki67)-PE (clone, SolA), CD4-Percpcy5.5 (clone, RM4-5), and CD8aα-Percpcy5.5 (clone, 53-6.7) were from BD Biosciences (San Diego, CA, USA). Anti-mouse CD45-Percpcy5.5 (clone, 30-F11), CD48-Allophycocyanin (APC)-Cy7 (clone, HM48-1), CD135-PE (clone, A2F10), CD34-Fluorescein isothiocyanate (FITC) (clone, RAM34), CD44-FITC (clone, IM7), CD3-PE-cy7 (clone, 145-2C11), T cell receptor gamma delta (TCRγ/δ)-APC (clone, GL3), Notch1-BV421 (clone, HMN1-12), Notch2-APC (clone, HMN2-35), and stem cell antigen 1 (Sca-1)-APC (clone, D7) were purchased from BioLegend (San Diego, CA, USA). CD117 (c-Kit)-PE-eFluor610 (clone, 2B8), CD25-PE (clone, PC61.5), and TCRβ-PE-eFluor610 (clone, H57-591) were purchased from eBioscience (San Diego, CA, USA).

### Cell co-culture

Mouse OP9-DL1 cells were seeded into 12-well plates (at 3 × 10^4^ cells/well), and the sink rate reached 80% overnight. The liver or bone marrow LSK cells were sorted and suspended in 2 ml of α-minimal essential medium (MEM) and added to the upper layer of OP9-DL1 cells supplemented with IL-7 (1 ng/mL) and FLT3-L (5 ng/mL). The IFN-γ-stimulated group was additionally supplemented with IFN-γ to a final concentration of 20 ng/ml. Fresh OP9-DL1 cells were seeded every 3 days. LSK cells were collected from the co-culture system and filtered into a new 50-mL centrifuge tube using a 40-μm cell strainer and centrifuged at 400 rcf for 5 min. The cell precipitate was resuspended in 2-mL α-MEM medium and seeded onto the fresh OP9-DL1 cells supplemented with 1 ng/mL IL-7 and 5 ng/mL FLT3-L. Cell mixtures were cultured in vitro at 37 °C in a 5% CO_2_ incubator. The co-cultured cells were harvested on day 7. Antibody staining and flow cytometry were performed on the harvested cells.

### Apoptosis assays

Liver and bone marrow LSK cells in the co-cultured system were harvested on day 14 and stained with 7-aminoactinomycin D (7-AAD) in an apoptosis detection kits (BD Biosciences).

### Quantitative real-time PCR

Total RNA was extracted from sorted liver or bone marrow LSK cells using an E.Z.N.A.MicroElute Total RNA Kit. (OMEGA, Atlanta, USA). The RNA concentration was quantified using a Nanodrop 2000 spectrophotometer (BioTek, Winooski, VT, USA). cDNA was generated using a FastQuant RT Kit (Tiangen Biotech CO. Ltd., Beijing, China). Quantitative real-time polymerase chain reaction (qPCR) was performed using a SYBR Green Supermix kit (Roche, Basel, Switzerland). Gene-specific primers were used as follows: *HES1* (encoding Hes family BHLH transcription factor 1): 5′-ACACCGGACAAACCAAAGAC-3′, 5′-ATGCCGGGAGCTATCTTTCT-3′; *HES5* (Hes family BHLH transcription factor 5): 5′-CAAGGAGAAAAACCGACTGC-3′, 5′-GGCTTTGCTGTGTTTCAGGT-3′; *Dll1* (delta like canonical notch ligand 1): 5′-CAGGACCTTCTTTCGCGTATG-3′, 5′-AAGGGGAATCGGATGGGGTT-3′; *Dll4* (delta like canonical notch ligand 4): 5′-TTCCAGGCAACCTTCTCCGA-3′, 5′-ACTGCCGCTATTCTTGTCCC-3′; *Jagged1* (jagged canonical notch ligand 1): 5′-CTACATACAGCATCTACATGC-3′, 5′-TCAGGCATGATAAACCCTAGC-3′; and *Actb* (beta actin): 5′-TGGAATCCTGTGGCATCCATGAAAC-3′, 5′-TAAAACGCAGCTCAGTAACAGTCCG-3′. The 2^−∆∆^CT (cycle threshold) equation was used to calculate the relative expression of target genes against that of *Actb* [33].

### γ-Secretase inhibitor (GSI) treatment

The GSI, LY-411,575 (a mixture of four diasteriomers of a small molecule inhibitor of γ-secretase), was synthesized as described previously [34]. Six-week-old male C57BL/6 J mice received a gavage of GSI (10 mg/kg/d dissolved in dimethyl sulfoxide (DMSO), resuspended in 50 mL of corn oil). Control mice were given a gavage of DMSO in corn oil. The expression levels of the downstream target genes *Hes1* and *Hes5* in the notch signal pathway were detected 1 week later to determine the blocking effect.

### Cytokine detection by enzyme-linked immunosorbent assay (ELISA)

Blood (100 μL) was collected from the normal mice or mice injected with plasmid plive-IFN-γ and left at room temperature for 30 min. The samples were centrifuged at 400 rcf for 15 min, and the serum supernatant was retained. The level of IFN-γ in serum was detected using an ELISA kit (Peprotech) in accordance with the manufacturers’ instructions.

### The JAK/STAT inhibitor ruxolitinib and cell culture

Ruxolitinib is a JAK1/2 inhibitor that can block the Janus kinase (JAK)/signal transducer and activator of transcription (STAT) signaling pathway. Ruxotinib was obtained from Selleck Chemicals (Houston, TX, USA), and stock solutions were prepared in DMSO. The sorted cells were incubated in the indicated concentrations of ruxolitinib (10 μM) for 1 h before stimulation. Cells were stimulated with 100 ng/ml IFN-γ for 30 min. Western blotting and qPCR were performed on the harvested cells.

### Western blotting

Bone-marrow-derived macrophages (BMDMs) were lysed directly into SDS sample buffer, and aliquots were run on 10% polyacrylamide gels using standard methods. Proteins were transferred onto nitrocellulose membranes, and specific proteins were detected by immunoblotting. Antibodies against phosphor-Y701-STAT1 (signal transducer and activator of transcription 1), total STAT1, and β-actin were obtained from Cell Signaling Technology (Danvers, MA, USA); HRP-conjugated secondary Abs were purchased from Beyotime (Jiangsu, China).

### BrdU incorporation assay

For bromodeoxyuridine (BrdU) labeling and staining, mice received an initial intraperitoneal injection of 3 mg of BrdU, followed by 3 days of BrdU in drinking water (1 mg/mL). BrdU incorporation into long-term hematopoietic stem cells (LT-HSCs) was detected using flow cytometry with an APC-BrdU flow kit (eBiosciences).

### Statistical analysis

Statistical analysis for pairwise comparisons was performed using Student’s *t* test for paired samples (data following a normal distribution), whereas that for multiple comparisons was performed by analysis of variance, when values followed normal distribution, or nonparametric tests, using GraphPad software (GraphPad Software, Inc., La Jolla, CA, USA). A threshold value of *P* < 0.05 was considered significant.

## Results

### IFN-γ inhibits the proliferation ability of HSPCs from the liver and bone marrow

**Fig. 1:**
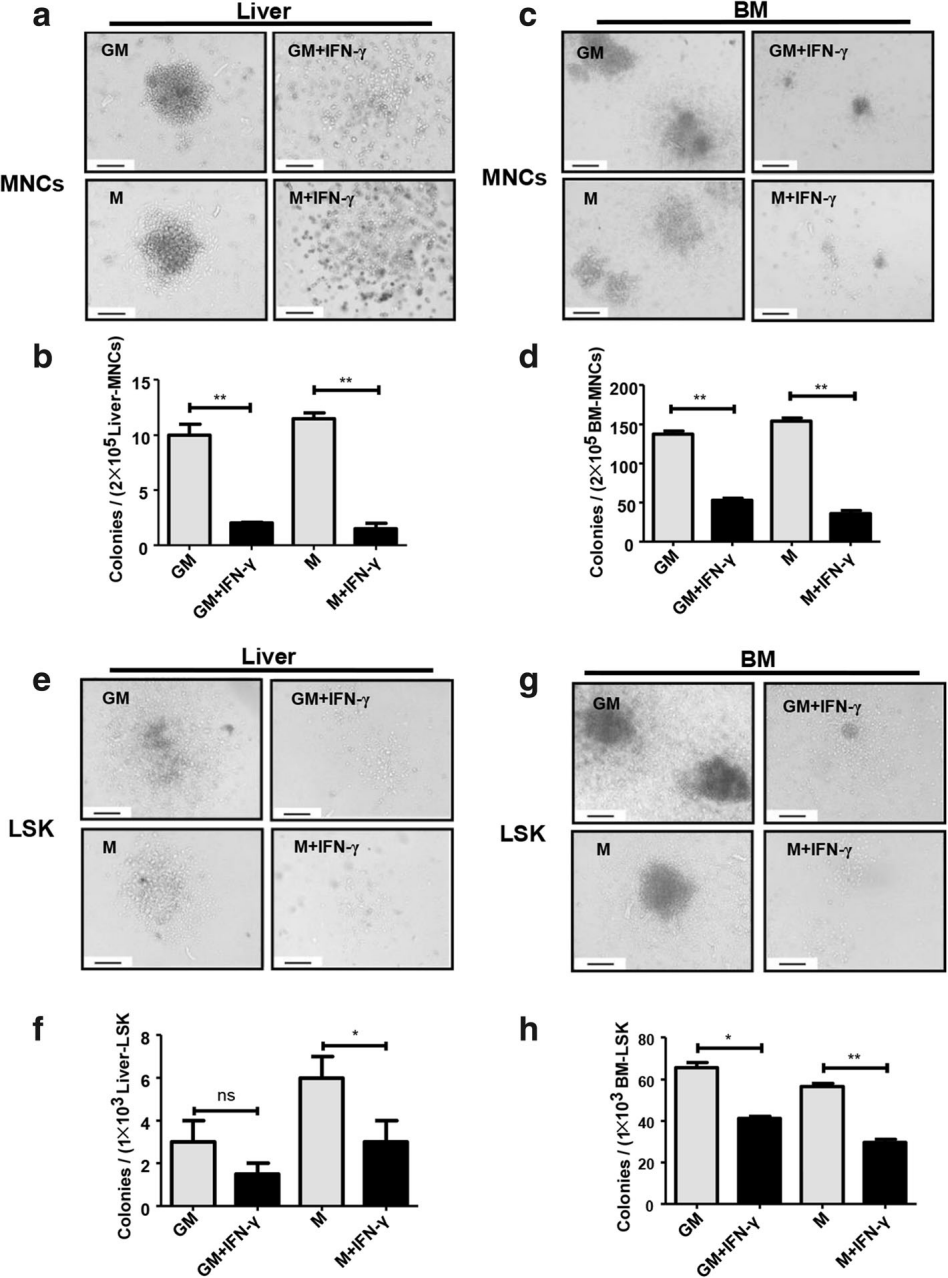
IFN-γ inhibits the colony-forming activity of HSCs of liver and bone marrow. Hematopoietic colony formation of the mononuclear cells (**a**, **c**) or LSK cells (**e**, **g**) from the adult liver (**a**, **e**) or bone marrow (**c**, **g**) (the picture shows a single colony in a well of 24-well cell culture plate). A total of 1 × 10^5^ bone morrow or liver mononuclear cells were freshly isolated from adult C57BL/6j mice, and 1 × 10^3^ bone marrow or liver LSK cells were sorted by FACS and plated into complete methylcellulose medium, with or without IFN-γ stimulation, and incubated for 10 to 14 days. The number of colonies (≥ 50 cells were defined as one clone) was counted under an inverted phase contrast microscope. GM-CFU: the added cytokines included SCF, IL-3, FLT-3-L, IL-7, and GM-CSF. M-CFU: the added cytokines included SCF, IL-3, FLT-3-L, IL-7, and M-CSF. Picture original magnification: × 200 (**a**, **c**, **e**, and **g**). Scale bar, 200 μm. Statistical analysis for the number of GM-CFUs and M-CFUs from MNCs or LSK cells of the BM and liver (*n* = 3–6) (**b**, **d**, **f**, and **h**). All colonies were counted in a well of 24-well cell culture plate. Bars represent the mean ± SEM of three independent experiments. *p < 0.05, **p < 0.01, ***p < 0.001

HSPCs have the ability to self-renew and differentiate. Previous studies have identified that IFN-γ impairs the proliferation of HSPCs in the BM [28, 32]. Therefore, we determined whether IFN-γ influenced the hematopoietic activity of liver HSPCs from MNCs. We isolated the liver and BM MNCs from C57BL/6 J mice, and cultured them for 14 days with a semi-solid culture medium supplemented with the supporting hematopoiesis-related cytokines SCF, IL-3, FLT-3 L, GM-CSF, and M-CSF. However, we found the number of colonies formed by liver MNCs was dramatically decreased in the IFN-γ-treated group compared with that in the unstimulated group on day 14 (Fig. 1a, b). Similarly, the number of colonies formed by BM MNCs in the IFN-γ-treated group also decreased. Notably, stimulation with IFN-γ reduced the clone formation ability of liver and BM MNCs (Fig. 1a–d).

MNCs contain limited numbers of hematopoietic stem cell precursors; therefore, the HSPCs (termed LSK: Lineage^−^Sca-1^+^c-Kit^+^) from liver and BM were sorted using FACS and then cultured in a semi-solid matrix to detect their colony formation activity. The numbers of colonies comprising macrophages and granulo-macrophages formed by liver LSK cells were decreased in IFN-γ-treated group compared with that in the unstimulated group on day 14, although the difference was not significant for the colonies of granulo-macrophages (Fig. 1e, f). The numbers of colonies comprising macrophages and granulo-macrophages formed by BM LSK cells in IFN-γ-treated group also decreased (Fig. 1g, h). These results indicated that liver LSK cells have the ability to form colonies, although this ability was weaker than that of BM LSKs. IFN-γ stimulation reduced both the numbers of colonies and the numbers of cells in each colony compared with those in the untreated group (Fig. 1e–h). These results demonstrated that IFN-γ inhibits the colony formation ability of both liver and BM LSKs.

**Fig. 2:**
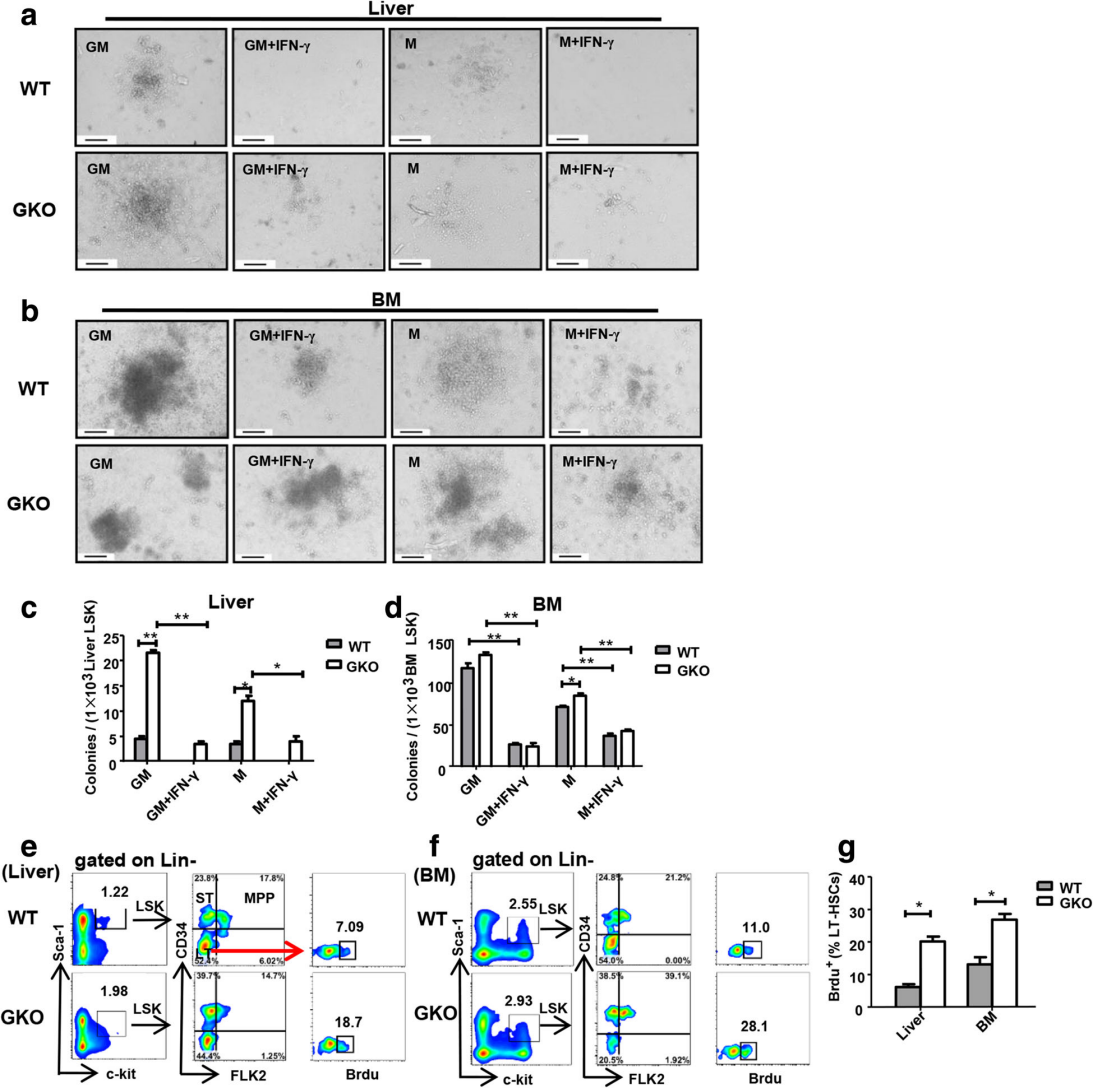
Knockout of IFN-γ promotes the proliferation ability of LSKs from liver and BM. Hematopoietic colony formation of LSK cells (**a**, **b**) from the adult liver (**a**) or bone marrow (**b**) (the picture shows a single colony in a well of 24-well cell culture plate). FACS was used to sort 1 × 10^3^ bone marrow or liver LSK cells from adult C57BL/6j mice and GKO mice and plated into complete methylcellulose medium with or without IFN-γ stimulation and incubated for 10 to 14 days. The number of colonies (≥ 50 cells were defined as one clone) was counted under an inverted phase contrast microscope. GM-CFU: the added cytokines included SCF, IL-3, FLT-3-L, IL-7, and GM-CSF. M-CFU: the added cytokines included SCF, IL-3, FLT-3-L, IL-7, and M-CSF. Picture original magnification: × 200 (**a**, **b**). Scale bar, 200 μm. Statistical analysis for the number of GM-CFUs and M-CFUs from LSK cells of the BM and liver (*n* = 3–6) (**c**, **d**). All colonies were counted in a well of a 24-well cell culture plate. Bars represent the mean ± SEM of three independent experiments. *p < 0.05, **p < 0.01, ***p < 0.001. The proportion of Lin^−^ c-kit^+^ sca-1^+^ (LSK) cells and LT-HSC, ST-HSC, and MPP cells gated from LSK cells and BrdU gated from LT-HSCs in the flow plot from the adult C57BL/6j mice and GKO mouse livers or BM (*n* = 5–10) (**e**, **f**). Statistical analysis of the percentage of BrdU cells gated from the LT-HSCs in the adult mouse liver and BM (**g**). Bars represent the mean ± SEM of three independent experiments. *p < 0.05

To further confirm the effects of IFN-γ on liver and BM HSPCs, we compared the colony-forming ability of LSKs from the liver and BM of IFN-γ-deficient mice (hereinafter referred to as GKO mice) and the C57BL/6 J mice (hereinafter referred to as wild-type (WT) mice). We found that liver LSKs from the GKO mice formed more colonies than those from WT mice (Fig. 2a, c). Similarly, BM LSKs from the GKO mice also formed more colonies than those from WT mice (Fig. 2b, d). The results showed that the colony formation ability of liver LSKs from GKO mice was stronger than of WT mice. In addition, stimulation with IFN-γ reduced the colony-forming ability of liver and BM LSKs from both WT and GKO mice (Fig. 2c, d). To further investigate the effect of IFN-γ on liver LSK cell proliferation. BrdU incorporation in LT-HSCs was detected using flow cytometry with an APC-BrdU flow kit. The results showed that BrdU could be incorporated into liver and BM LT-HSCs, with more BrdU being incorporated into liver and BM LT-HSCs from both GKO mice than into those of WT mice (Fig. 2e–g). These results indicated that IFN-γ inhibits the proliferation ability in liver and BM LSKs.

### IFN-γ impairs the differentiation of LT-HSCs into ST-HSCs and MPPs

**Fig. 3:**
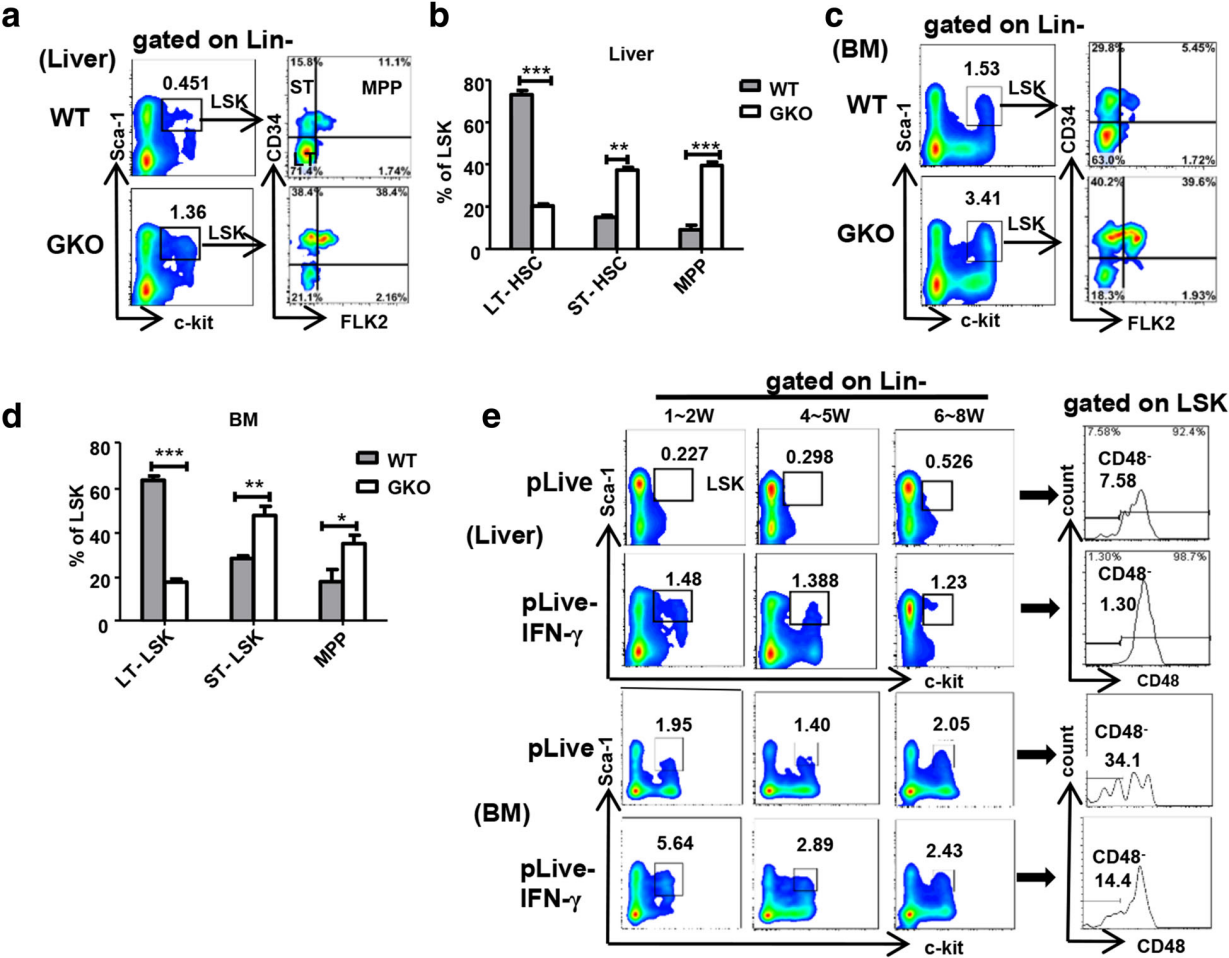
IFN-γ impacts the differential progress of LT-HSC to ST-HSC and MPPs in the liver and BM. The proportion of Lin^−^ c-kit^+^ sca-1^+^ (LSK) cells and LT-HSC, ST-HSC, and MPP cells in the flow plot was gated from LSK cells from the adult C57BL/6j mice and GKO mouse liver or BM (*n* = 5–10) (**a**, **c**). Statistical analysis of the percentage of LT-HSC, ST-HSC, and MPP cells gated from the LSK cells in the adult mice liver and BM (**b** and **d**). *p < 0.05. **e** Flow cytometry plots showing the percentage of LSKs among the Lin^−^ cells and CD48^−^ cells gated from LSK cells from the bone marrow or liver of adult mice after hydrodynamic injected with pLive or pLive-IFN-γ plasmid for 1–8 weeks. (*n* = 5–10). Bars represent the mean ± SEM of three independent experiments

We next assessed whether IFN-γ could affect differentiation of liver and BM HSPCs. Adult mouse BM hematopoietic stem cells can be divided into long-term hematopoietic stem cells (LT-HSCs: Lin^−^Sca-1^+^c-Kit^+^CD34^−^FLK2^−^), short-term hematopoietic stem cells (ST-HSCs: Lin^−^Sca-1^+^c-Kit^+^CD34^+^FLK2^−^), and multipotent progenitors (MPPs: Lin^−^Sca-1^+^c-Kit^+^CD34^+^FLK2^+^). They are all included in the group of LSK cells and develop in the order LT-HSCs to ST-HSCs to MPPs. Previous reports indicated that IFN-γ affected not only the self-renewal ability of HSCs but also their fate [28]. To determine whether IFN-γ impaired the differentiation of liver and BM HSPCs, we compared the proportions of different stages of hematopoietic stem cells from GKO and WT mice. GKO mice had higher proportions of LSK cells than WT mice both in the liver and BM (Fig. 3a, c). Furthermore, the proportions of LT-HSCs cells to total LSKs from liver and BM in GKO mice were decreased compared with those in the WT mice, while the proportions of ST-HSCs and MPPs increased (Fig. 3a–d). These results indicated that IFN-γ inhibits the differentiation process from LT-HSCs to ST-HSCs and MPPs.

We next established an IFN-γ overexpression mouse model using hydrodynamic injection of plasmid pLive-IFN-γ. The serum levels of IFN-γ were detected using ELISA. The mice could overexpress IFN-γ stably at 5000 pg/mL after 4 weeks (Additional file 1: Figure S1a). The level of serum alanine transaminase (ALT) was in the normal range after 1 week (Additional file 1: Figure S1b), which suggested that hydrodynamic injection of pLive-IFN-γ did not cause liver injury. These results demonstrated the successful establishment of the IFN-γ overexpression mouse model. We then compared the proportions of LSK cells to total HPSCs from the liver and BM in the IFN-γ overexpression mice from week 1 to week 8. The LSK ratio from liver and BM in IFN-γ overexpression mice tended to be higher than that for the control group after 2 weeks (Fig. 3e). Interestingly, the proportions of liver LSK cells from the IFN-γ overexpression mice increased compared with that in the control mice. It has been reported that IFN-γ stimulates SCA-1 expression [32, 35–39]. In this study, we observed that there was indeed a significant upregulation of SCA-1 levels in the bone marrow and liver after injection of pLive-IFN-γ at week 1 (Additional file 1: Figure S2a, b). This upregulation indicated that the increase in the proportion of liver LSKs might be caused by upregulation of SCA-1 expression. It has been reported that the repopulating activity was found to reside only in the CD48^−^ LSK cell population and that the expression of CD48 predicts the loss of stemness characteristics [40]. In other words, CD48^−^ LSK cells have strong self-renewal and differentiation abilities. Thus, we next focused on the expression of CD48 on liver and BM LSK cells in the IFN-γ overexpression mice. The results showed that CD48 expression on both liver and BM LSKs from IFN-γ overexpression mice decreased compared with that in the control group (Fig. 3e), which indicated that IFN-γ led to a decrease in the number of liver stem cells with strong stemness characteristics. These results suggested that IFN-γ inhibits the differentiation process of liver and BM HSPCs.

### IFN-γ inhibits the differentiation of liver T precursor cells in vivo

**Fig. 4:**
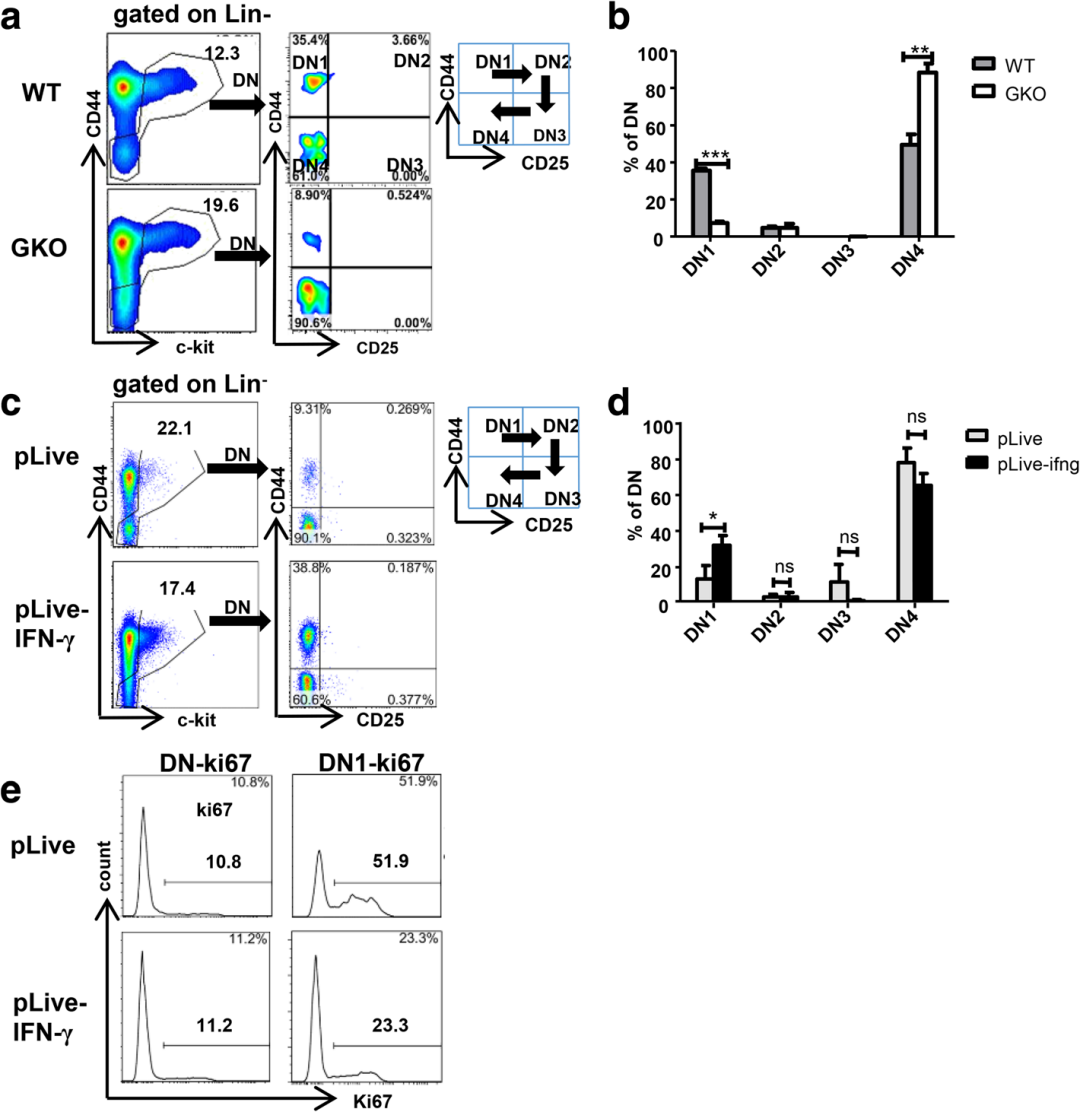
IFN-γ inhibits the development of liver T progenitor cells in vivo. The proportion of T progenitor cells (the whole range of DN cells) and DN1, DN2, DN3, and DN4 cells in the flow plot was gated from DN cells from the adult C57BL/6j mice and GKO mouse liver (**a**); adult mice overexpressing IFN-γ and the control mice (**c**) (*n* = 5–10). **b**, **d** Statistical analysis for the percentage DN1, DN2, DN3, and DN4 cells gated from the whole DN cells in the adult C57BL/6j mice and GKO mouse liver (**b**) and the adult mice overexpressing IFN-γ and the control mice (**d**). Bars represent the mean ± SEM of three independent experiments. *p < 0.05, **p < 0.01, ***p < 0.001. **e** Flow cytometry plots showing the expression of Ki67 on total DN cells and DN1 cells from the adult mouse liver after hydrodynamic injection with plasmid pLive or pLive-IFN-γ for 8 weeks

HSPCs can differentiate into erythroid progenitor cells, myeloid progenitor cells, and lymphoid progenitor cells. However, the effect of IFN-γ on this differentiation process is unknown. Studies have shown that IFN-γ can directly impair the differentiation of CLPs into T cells both in vitro and in vivo [41, 42]. Therefore, we determined the effect of IFN-γ on the differentiation of lymphoid progenitor cells by assessing T precursor cells differentiation in GKO and IFN-γ overexpression mice. We compared the proportion of T-cell progenitor subsets in liver of GKO mice and WT mice. The results showed that the proportion of total double-negative (DN) cells in GKO mouse increased compared with those in the WT mice; however, we observed a decreased proportion of the DN1 cell subset, while the proportion of the DN4 cell subset obviously increased in the GKO mouse (Fig. 4a, b). These results suggested that IFN-γ interferes with the differentiation of DN1 cells into DN4 cells.

We further compared the proportion of the T progenitor cells DN1 to DN4 cells in the liver of IFN-γ overexpression mice and the control mice. The proportion of total DN cells in the liver of the IFN-γ overexpression mice was decreased slightly compared with that of the control mice. Interestingly, we found that the proportion of DN1 cell subset increased and the proportion of DN4 cell subset decreased in the IFN-γ overexpression compared with that of the control mice (Fig. 4c, d). These results suggested that overexpression of IFN-γ arrests T progenitor cells in the DN1 phase, which interferes with the differentiation of DN1 cells into DN4 cells. We then assessed the expression of Ki67 on liver T progenitor cells. The expression of Ki67 on liver DN1 cells from the IFN-γ overexpression mice decreased significantly, while there was no difference in Ki67 expression in the total DN cells compared with that in the WT mice (Fig. 4e). These results showed that IFN-γ might arrest T precursor cells in the DN1 phase by reducing the proliferative capacity of DN1 cells.

### IFN-γ affects the differentiation of liver T precursor cells and γδT cells in vitro

**Fig. 5:**
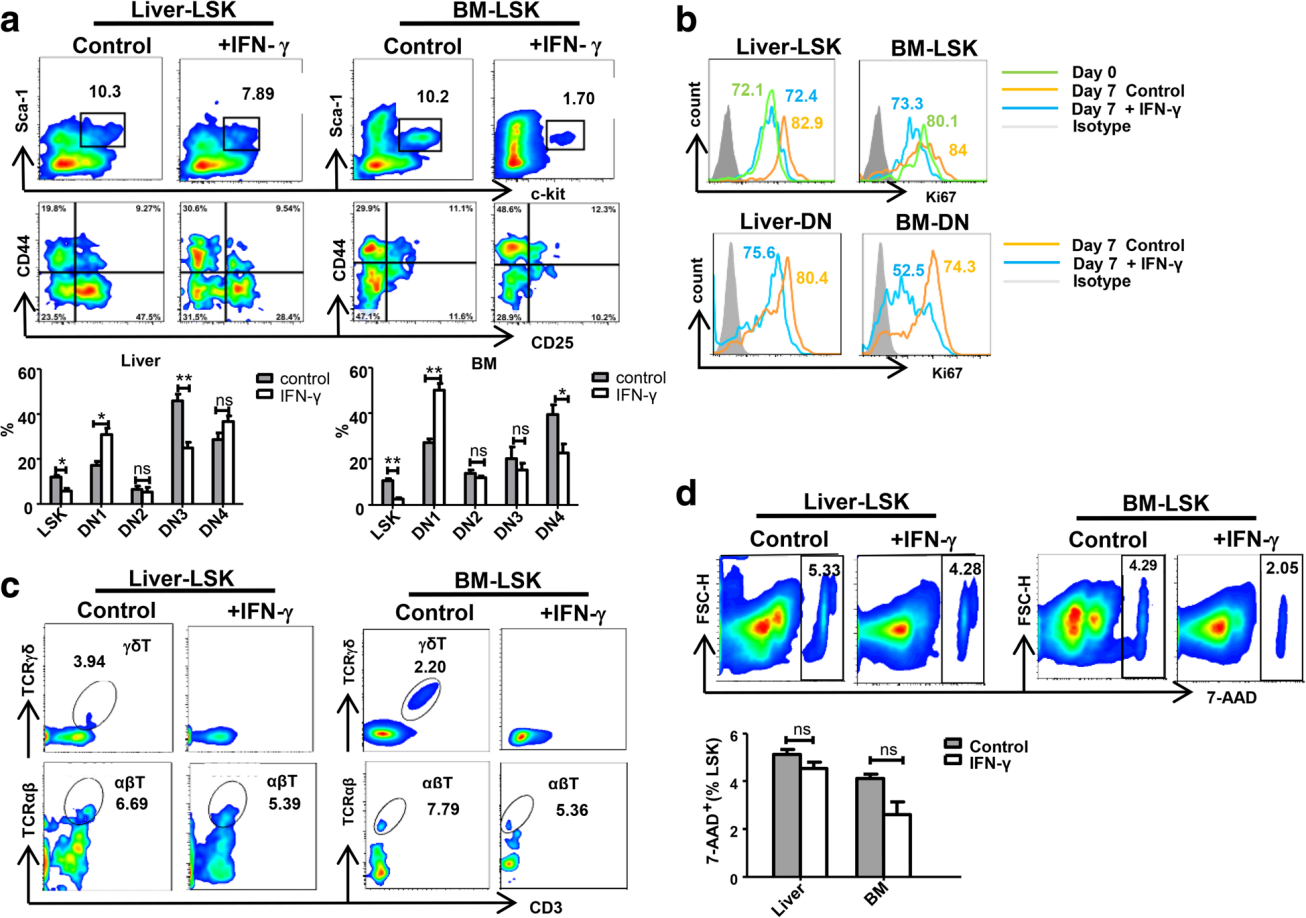
IFN-γ impacts the differentiation rate from LSK cells to T progenitor cells and γδT cells. **a** The proportion of LSK cells and T progenitor cells (DN1, DN2, DN3, and DN4 cells) in the flow plot was gated from Lineage^−^ cells and the expression of Ki67 on total LSK cells and DN cells after the adult mice liver or BM LSK cells were co-cultured with OP9-DL1 cells, with or without IFN-γ stimulating at days 7 (*n* = 5–10). **b** The expression of Ki67 on total LSK cells and DN cells after the adult mouse liver or BM LSK cells were co-cultured with OP9-DL1 cells, with or without IFN-γ stimulation at day 0 and day 7 (*n* = 5–10). **c** The proportion of γδT cells and αβT cells in the flow plot after the adult C57BL/6j mice liver or BM LSK cells were co-cultured with OP9-DL1 cells, with or without IFN-γ stimulating at day 21. **d** Apoptosis levels of liver LSK cells and BM LSK cells in the co-cultured system were detected using 7-AAD at day 21

Previous experimental results showed that IFN-γ may inhibit the differentiation of T precursor cells in vivo. To observe whether IFN-γ affects differentiation of T precursor cells in vitro, we sorted out LSK cells from the liver and BM and co-cultured them with OP9-DL1 cells together with cytokines IL-7 and FLT-3 ligand to observe the differentiation process. The results showed that the LSK cells from the liver and BM had differentiated into DN2 and DN3 cells at day 7, and there were still LSK cells in the co-culture system. Interestingly, the proportion of liver and BM LSK cells decreased after stimulation with IFN-γ. In addition, the number of liver DN1 cells increased, while the number of differentiated DN3 cells decreased after stimulation with IFN-γ. The number of BM DN1 cells also increased while the number of differentiated DN4 cells decreased after stimulation with IFN-γ (Fig. 5a). The expression of Ki67 on LSK cells and DN cells also decreased after stimulation with IFN-γ (Fig. 5b). The results further supported the view that IFN-γ inhibits the differentiation of liver LSK cells into T progenitor cells.

To further investigate whether IFN-γ affects the developmental process of T cell progenitor cells into mature αβT and γδT cells [43, 44], we extended the time of co-culturing to 21 days. We found that in the presence of IFN-γ, the liver and BM LSK cells could hardly differentiate into γδT cells, while IFN-γ had no significant effect on the differentiation of liver and BM LSK cells into αβT cells. (Fig. 5c). Moreover, the number of cells in co-culture system stimulated with IFN-γ was significantly reduced. To investigate whether IFN-γ inhibited differentiation or caused cell death or apoptosis in the culture system, we used 7-AAD to detect apoptosis of the sorted cells in culture systems. The results showed no difference in the proportion of apoptotic (7-AAD^+^) T cells in the co-culture system compared with that in the control group (< 5%) (Fig. 5d). The results indicated that IFN-γ-induced impairment of the development of hematopoietic stem cells into γδT cells does not involve cell apoptosis.

### The molecular mechanism of IFN-γ’s effects on the differentiation of liver and bone marrow HSPCs

**Fig. 6:**
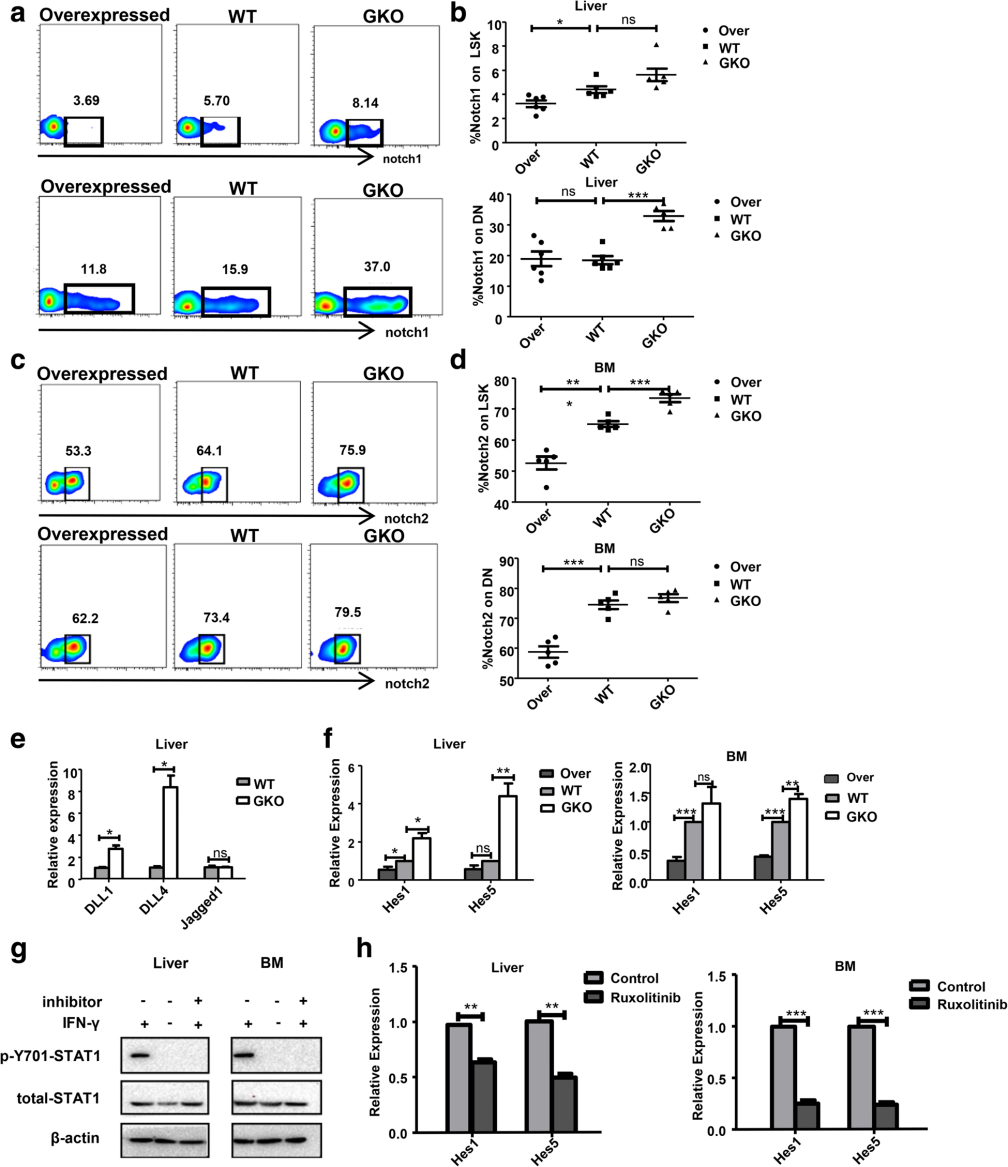
IFN-γ inhibits the activation of notch signaling. **a** Flow cytometry plots showing the expression of notch1 on the LSK cells and DN cells of mice, GKO mice, and IFN-γ overexpressing mouse livers (**a**) and the expression of notch2 on LSK cells and DN cells of C57BL/6j mice, GKO mice, and IFN-γ overexpressing mouse BM (**c**) (*n* = 5–10). **b**, **d** Statistical analysis of the expression of notch1 (**b**) and notch2 (**d**) on LSK cells and DN cells of mice, GKO mice, and IFN-γ overexpressing mouse livers and BM. Bars represent the mean ± SEM of three independent experiments. *p < 0.05, **p < 0.01, ***p < 0.001. **e** The gene expression level of ligands of notch signal on the LSEC in the WT and GKO mice. Bars represent the mean ± SEM of three independent experiments. *P < 0.05. **f** The gene expression level of Notch downstream targets *Hes1* and *Hes5* on the LSK cells in the liver and BM of IFN-γ overexpressing mice, WT, and GKO mice. **g** The LSK cells of the liver and BM were sorted from WT mice and incubated in the indicated conditions of ruxolitinib (10 μM) for 1 h before stimulation with 100 ng/ml IFN-γ for 30 min. Levels of β-actin as well as total and phospho-Tyr701 STAT1 were determined using immunoblotting. **h** As in (**g**), the gene expression level of *Hes1* and *Hes5* on the LSK cells of the liver and BM in ruxolitinib-treated mice and the control mice were detected. Bars represent the mean ± SEM of three independent experiments. *p < 0.05, **p < 0.01, ***p < 0.001

IFN-γ is widely viewed as a negative regulator of HSPCs’ self-renewal and differentiation [17]. However, the underlying mechanism of IFN-γ effects on hematopoiesis is unknown. It has been established that IFN-γ signaling acts downstream of notch signaling in HSC development and that IFN-γ could disrupt the expression of Notch target genes *HES1* and *HEY1* in patients with autoimmune diseases [45]. Notch signaling also plays crucial roles during the establishment of HSC fate [46–49]. Therefore, we determined whether the inhibitory effect of IFN-γ on liver HSPCs acts via notch signaling. First, we observed that liver LSK cells and T precursor cells expressed notch1, and the expression of notch1 on GKO mice LSK cells and DN cells was higher than that on WT mice, whereas its expression on IFN-γ overexpression mice was reduced (Fig. 6a, b). Unlike the liver, BM LSK cells and T precursor cells express notch2. Compared with that in WT mice, the expression of notch2 on GKO mice was higher, whereas the expression in IFN-γ overexpression mice was lower (Fig. 6c, d). Similarly, the expression of notch2 on liver LSK cells and T precursor cells in GKO mice increased (Additional file 1: Figure S3a). However, BM LSK cells and T precursor cells did not express notch1 (Additional file 1: Figure S3b).

Furthermore, we examined the expression of notch ligands on liver sinusoidal endothelial cells of WT and GKO mice. The expression levels of DLL1 and DLL4 increased on sinusoidal endothelial cells of GKO mice compared with those in WT mice; however, there was no significant difference in the expression of Jagged1 between WT and GKO mice (Fig. 6e). Subsequently, we found that the expression of *Hes1* and *Hes5* in GKO mice liver and BM LSK cells was increased compared with that from WT mice, whereas the expression of *HES1* and *HES5* decreased in IFN-γ overexpression mice (Fig. 6f). These results indicated that IFN-γ might inhibit the activation of notch signaling. Mechanistically, IFN-γ transmits signals by inducing the formation of STAT-1 homologous dimer to modulate the JAK-STAT pathway [50]. To explore if the effect of IFN-γ on notch signaling acts via JAK-STAT pathway, we used the JAK1/2 inhibitor ruxolitinib to block the JAK-STAT signaling pathway. The blocking effect was verified using western blotting (Fig. 6g). The levels of molecules of the notch signaling pathway, *HES1* and *HES5*, in liver and BM LSK cells were detected. We found that inhibition of JAK/STAT with ruxolitinib significantly decreased the levels of *HES1* and *HES5* in both liver and BM LSK cells (Fig. 6h). These results implied that IFN-γ might inhibit the activation of notch signaling via the JAK/STAT signaling pathway.

**Fig. 7:**
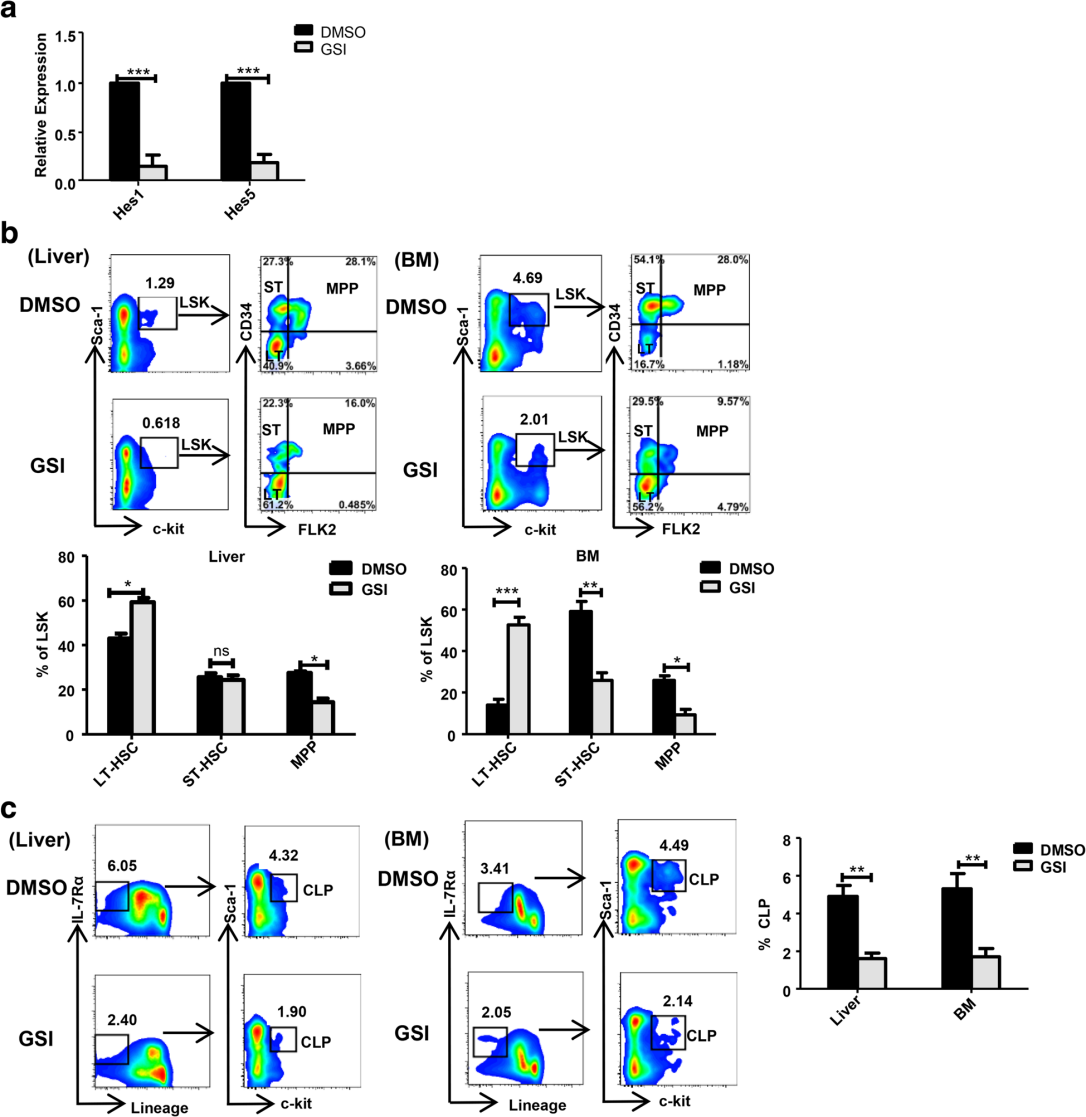
Notch signaling impacts the differentiation of liver and BM hematopoietic stem cells. **a** Six-week-old male mice were given a gavage dose of GSI and DMSO for 1 week, and the gene expression level of *HES1* and *HES5* on the LSK cells of the liver in the GSI-treated mice and the control mice were detected. **b** The proportions of LSK and LT-HSC, ST-HSC, and MPP cells in the flow plot were gated from LSK cells from the GSI-treated mice and the control mice livers or BM (*n* = 5–10). **c** The proportion of CLP cells in the flow plot was gated from Lin^−^ IL-7Rα^+^ cells from the GSI-treated mice and the control mice livers or BM (*n* = 5–10). Bars represent the mean ± SEM of three independent experiments. *p < 0.05, **p < 0.01, ***p < 0.001

To further investigate whether IFN-γ inhibits the differentiation of HSPCs by interfering with the activation of notch signaling, we examined the proportion of differentiated liver and BM HSPCs before and after blocking notch signaling with the γ-secretase inhibitor (GSI), which blocks notch intracellular domain (NICD) release from the plasma membrane. Compared with those in the control group, the expression levels of *Hes1* and *Hes5* in GSI-treated mice LSK cells were significantly decreased, suggesting that notch signaling was successfully blocked by GSI (Fig. 7a). Subsequently, we found that the proportion of LSK cells in GSI-treated mice was reduced, and the proportion of LT-HSCs was significantly increased, while the proportion of ST-HSCs and MPP cells was decreased, which demonstrated that the development of HSPCs from LT-HSCs to ST-HSCs and MPPs was inhibited by blocking the notch signaling pathway (Fig. 7b). Similarly, the proportion of CLP cells in GSI-treated mice was also reduced, which further suggested that the differentiation of HSPCs was inhibited by blocking the notch signaling pathway (Fig. 7c). These results suggest that IFN-γ inhibits the differentiation of HSPCs by interfering with notch signaling, revealing the possible molecular mechanism by which IFN-γ affects the differentiation of liver and BM HSPCs.

## Discussion

Bone marrow is the most important hematopoietic organ after birth and can replenish all blood cell types throughout life [4–6]. Previously, studies reported that a small number of HSCs which exist in the adult liver possess hematopoietic-reconstitution ability [10, 14, 15]. The hematopoietic microenvironment is critical to support and regulate the self-renewal, expansion, and differentiation of HSPCs in hematopoietic tissues [12]. The effect of IFN-γ on BM hematopoietic system has been studied [21, 28, 32, 41, 42, 51–55]. However, whether IFN-γ regulates adult liver hematopoiesis remains unclear. In the present study, we confirmed that IFN-γ inhibited the colony-forming activity and the proliferation ability of liver HSPCs. In addition, we found that IFN-γ impaired the differentiation of adult liver HSPCs including the differentiation of LT-HSCs into ST-HSCs and MPPs, the differentiation of LSK cells into T progenitor cells, and the differentiation of T progenitor cells into γδT cells. Importantly, this is the first report that IFN-γ inhibits the development of liver T cell progenitors into γδT cells. Furthermore, we suggested a molecular mechanism whereby IFN-γ impairs HSPC proliferation and differentiation. We proposed that IFN-γ might act via the JAK/STAT signaling pathway to inhibit the activation of notch signaling, thereby inhibiting the differentiation of adult mouse liver and BM hematopoietic stem cells.

It is important to compare the differences between liver HSPCs and BM HSPCs in terms of their response to IFN-γ. In the present study, we found that IFN-γ inhibited the colony formation ability of both adult liver and BM; however, the degree of inhibition was different. The inhibitory effect of IFN-γ on the colony formation ability of liver LSK cells was weaker than that of BM LSK cells. Specifically, IFN-γ inhibited both the M-CFU and GM-CFU of BM LSK cells significantly. IFN-γ also inhibited the M-CFU and GM-CFU of liver LSK cells, but there was no significant difference for the GM-CFUs from liver LSKs. Moreover, in terms of IFN-γ-mediated inhibition of HSPC differentiation, the inhibition effect in the liver was weaker than that in the BM, especially for the differentiation of LSK cells into T progenitor cells, which was confirmed by the results of co-culture in vitro. In addition to IFN-γ, the hematopoietic system can be influenced by other proinflammatory cytokines, such as tumor necrosis factor α, granulocyte colony-stimulating factor, and IFN-α [17]. Whether the other proinflammatory cytokines have different influences on liver and BM HSPCs requires further study. Understanding the factors involved in the differentiation and proliferation of liver hematopoietic precursors could be a very important tool to reveal the dynamics of organ development and how the liver maintains cellular populations with unique characteristics throughout life.

IFN-γ has been widely viewed as a negative regulator of HSPC self-renewal and differentiation [17]. However, the underlying mechanism of IFN-γ in hematopoiesis has not been determined. In adult hematopoiesis, notch is a very efficient promoter of T cell differentiation [56, 57]. In this study, we found IFN-γ might impair the hematopoietic differentiation in the liver and BM by inhibiting notch signaling. Consistent with a previous report [45], we observed that IFN-γ impaired the activation of notch signaling of both liver and BM HSPCs. However, there was no obvious difference in the expression of Jagged1 on liver sinusoidal endothelial cell of WT mice compared with that in GKO mice. We speculated that IFN-γ might mainly affect the expression levels of DLL1 and DLL4, but has no significant effect on the expression of Jagged1. Mechanistically, IFN-γ transmits signals by inducing the formation of the STAT-1 homodimer to modulate the JAK-STAT pathway. To explore if the effect of IFN-γ on notch signaling acts via the JAK-STAT pathway, we used the JAK1/2 inhibitor ruxolitinib to block the JAK-STAT signaling pathway. Inhibition of JAK/STAT using ruxolitinib significantly decreased the expression of *HES1* and *HES5* in both liver and BM LSK cells. These results implied that IFN-γ might inhibit the activation of notch signaling through the JAK/STAT signaling pathway. Subsequently, we blocked notch signaling using a GSI [58–60] and found that the differentiation of liver and BM LT-HSCs into ST-HSCs and MPPs and the differentiation of LSK cells into CLPs were also inhibited. This result agreed with previous findings, in which whole BM cells from IFN-γ-treated mice showed reduced engraftment and ex vivo IFN-γ-expanded LSK cells failed to engraft [26]. However, how IFN-γ inhibits the activation of notch signaling requires more research.

Studies have found that BM hematopoietic arrest during lymphocytic choriomeningitis virus (LCMV) infection and in aplastic anemia (AA) is related to the production of IFN-γ [29, 40]. In patients with AA, IFN-γ is overproduced by auto-reactive T lymphocytes and is associated with altered HSPC distribution [31, 61], which may subsequently induce the progression of AA. Usually, the symptoms of patients with AA can be relieved using anti-IFN-γ antibodies [55, 62, 63]. IFN-γ is also associated with the hematopoietic suppression observed in patients with Fanconi anemia [22, 64]. From a therapeutic perspective, anti-IFN-γ antibodies improve the regenerative capacity of hematopoietic progenitors derived from patients with AA [26, 63]. Moreover, in a mouse model of T cell-induced BM failure, characterized by severe pancytopenia and BM hypoplasia, treatment with anti-IFN-γ enhanced the survival rate [24, 55, 65–67], which suggested the underlying mechanism of hematological malignancies involving liver HSPCs. The present study demonstrated, for the first time, that IFN-γ affects the hematopoietic function and differentiation of liver HSPCs. These findings not only provided experimental evidence for the differentiation and regulation of liver hematopoietic stem cells, but also will encourage the development of therapy to treat related diseases, such as viral infection and aplastic anemia, in which the bone marrow function is insufficient. The findings will also be useful to suggest strategies for the better use of liver hematopoietic stem cells in transplantation immunity.

## Conclusions

The present study demonstrated that IFN-γ inhibited the proliferation ability of liver-derived HSPCs. IFN-γ inhibited the differentiation of LT-HSCs to ST-HSCs and MPPs, and the differentiation of LSKs to γδT cells. We proposed that IFN-γ might act via the JAK/STAT signaling pathway to inhibit the activation of notch signaling, thereby impairing the differentiation of adult mouse liver and BM hematopoietic stem cells. These findings provide important insights into liver extramedullary hematopoiesis and the regulation of liver HSPCs and will be particularly important to develop therapies to treat hematological malignancies and diseases.

## Additional file

Additional file 1: Supplementary methods and results. **Figure S1.** Expression of IFN-γ in the serum of mice hydrodynamically injected with the pLive-IFN-γ plasmid. **Figure S2.** The expression of Sca-1 was induced in Lin^−^c-kit^+^ cells after hydrodynamic injection of the pLive-IFN-γ plasmid. **Figure S3.** The expression of notch1 or notch2 on the LSK and DN cells in the liver and BM of over-expressed IFN-γ mice, WT, and GKO mice. **Figure S4.** Genotype identification of IFN-γ deficient mice. (DOC 3432 kb)

## Data Availability

The data used to support the findings of this study are included within the article.

## Abbreviations

7-AAD: 7-Aminoactinomycin D
AA: Aplastic anemia
ALT: Alamine aminotransferase
BM: Bone marrow
CFU-GM: Colony-forming units-granulocyte/macrophage
CLP: Common lymphoid progenitor cells
DN: CD4^−^CD8^−^ cells
GKO: IFN-γ-deficient mice
GSI: γ-Secretase inhibitor
HSPCs: Hematopoietic stem and progenitor cells
Lin^−^: Mature lineage negative
LSK: Lineage^−^Sca-1^+^c-Kit^+^
LT-HSC: Long-term hematopoietic stem cells
MNCs: Mononuclear cells
MPP: Multipotent progenitor
qRT-PCR: Quantitative reverse-transcription polymerase chain reaction
SCA-1: Stem cell antigen-1
SCF: Stem cell factor
ST-HSC: Short-term hematopoietic stem cells
WT: Wild-type
