# Supplementary material for: Interferon gamma inhibits the differentiation of mouse adult liver and bone marrow hematopoietic stem cells by inhibiting the activation of notch signaling
Source: Stem Cell Res Ther. 2019 Jul 16;10:210. doi: 10.1186/s13287-019-1311-0 (PMC6636148; doi:10.1186/s13287-019-1311-0)
Supplement: Supplementary file 1 — Supplementary methods and results. Figure S1. Expression of IFN-γ in the serum of mice hydrodynamically injected with the pLive-IFN-γ plasmid. Figure S2. The expression of Sca-1 was induced in Lin−c-kit+ cells after hydrodynamic injection of the pLive-IFN-γ plasmid. Figure S3. The expression of notch1 or notch2 on the LSK and DN cells in the liver and BM of over-expressed IFN-γ mice, WT, and GKO mice. Figure S4. Genotype identification of IFN-γ deficient mice. (DOC 3432 kb) [file 13287_2019_1311_MOESM1_ESM.doc]

**Supplement methods**

**Serum alanine aminotransferase activity assays**

Alanine aminotransferase (ALT) activity in serum was quantified using a commercially available kit (Jian Cheng, Nanjing, China) at the indicated times after injection.

**Genotype identification of IFN-γ-deficient mice**

Claws of mice (to be identified mice and WT mice) were cut and placed in Eppendorf tubes with 250 μL proteinase K buffer and boiled for 10 min. After cooling to room temperature, 5μL of proteinase K was added,the tubes were inverted, and incubated overnight at 55 °C. Thereafter, the samples were briefly vortexed and boiled for 10 minutes. The samples were centrifuged at 1600 rcf at 4 °C and the supernatant was used as a template for PCR amplification. The amplified cDNA was then subjected to agarose gel electrophoresis.

Amplification procedure:

| Step | Temp. °C | Time | Notes |
| --- | --- | --- | --- |
| 1 | 94 | 2 min |  |
| 2 | 94 | 20 sec |  |
| 3 | 65 | 15 sec |  |
| 4 | 68 | 10 sec |  |
| 5 |  |  | Repeat steps 2–4 for 10 cycles |
| 6 | 94 | 15 sec |  |
| 7 | 60 | 15 sec |  |
| 8 | 72 | 10 sec |  |
| 9 |  |  | Repeat steps 6–8 for 28 cycles |
| 10 | 72 | 2 min |  |
| 11 | 10 | hold |  |

**Supplementary figure**

**
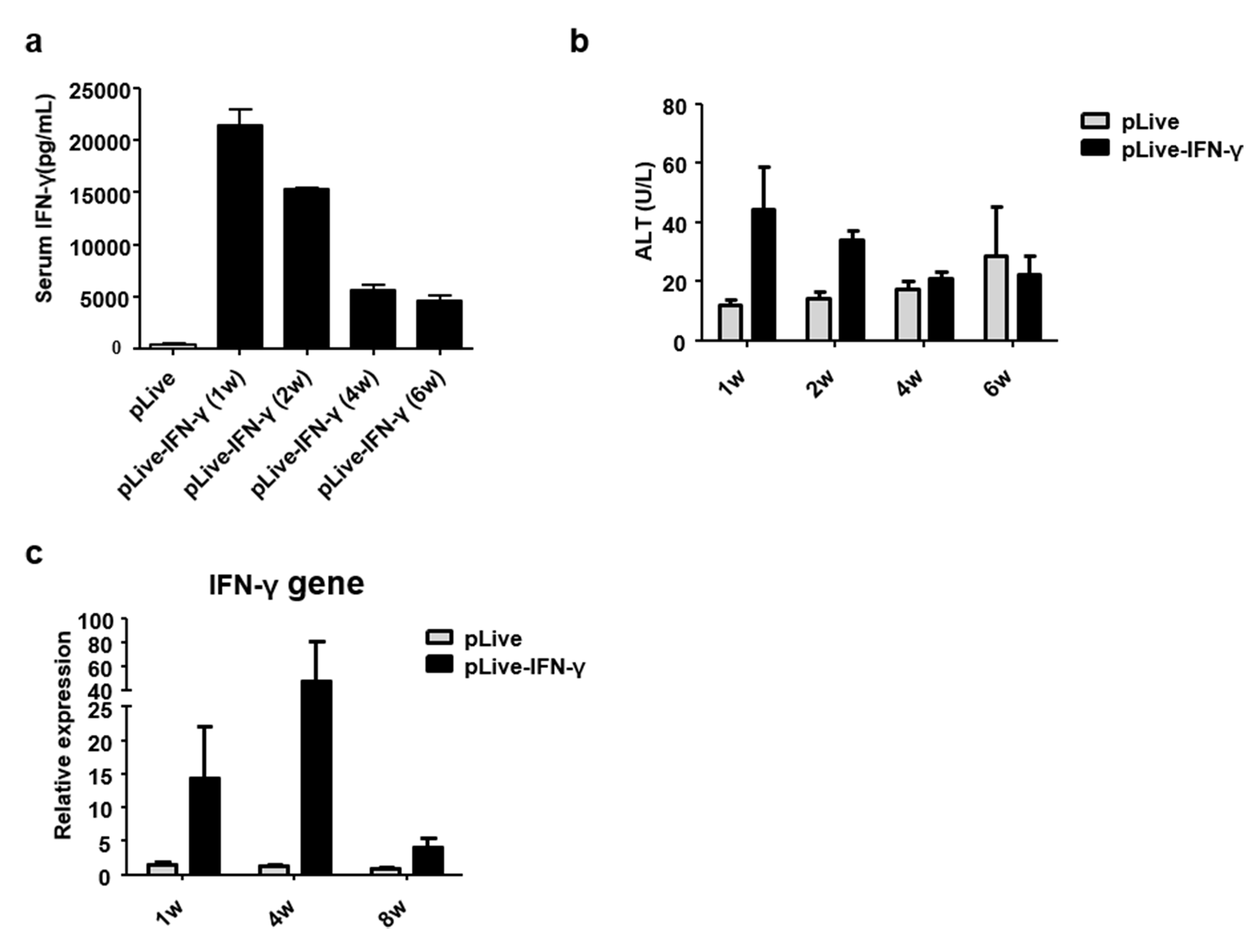
**

**Figure S1: Expression of IFN-γ in the serum of mice hydrodynamically injected with the pLive-IFN-γ plasmid.**

Mice were hydrodynamically injected with the pLive-IFN-γ plasmid, and the control mice were hydrodynamically injected with the pLive plasmid. (a) Concentration of serum IFN-γ was detected at week 1, 2, 4, and 6 using ELISA. (b) The alanine aminotransferase (ALT) activity was detected at week 1, 2, 4, 6. (c) The gene expression of *Ifng* in liver tissue was detected at week 1, 4, and 8. IFN-γ, interferon gamma; *Ifng*, interferon gamma gene/mRNA; ELISA, enzyme-linked immunosorbent assay.

**
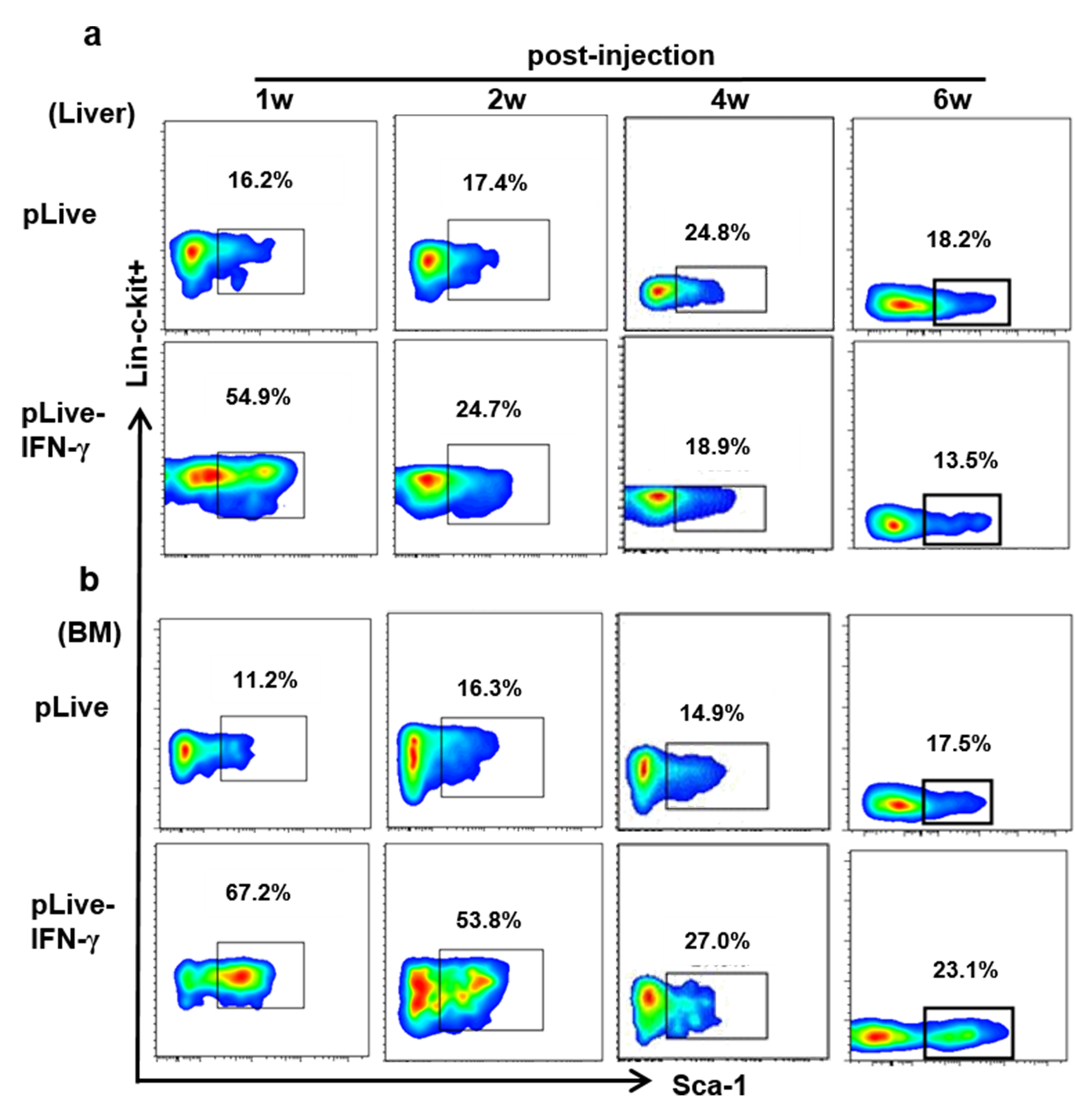
**

**Figure S2: The expression of Sca-1 was induced in Lin^-^c-kit^+^ cells after hydrodynamic injection of the pLive-IFN-γ plasmid.**

Mice were hydrodynamic injected with the pLive-IFN-γ plasmid, and the control mice were hydrodynamic injected with the pLive plasmid. Representative flow cytometry plot of the expression of Sca-1 on Lin^-^c-kit^+^ cells in the liver (a) and BM (b) at weeks 1–6 after injection. SCA-1, Spinocerebellar Ataxia 1; IFN-γ, interferon gamma; BM, bone marrow.

**
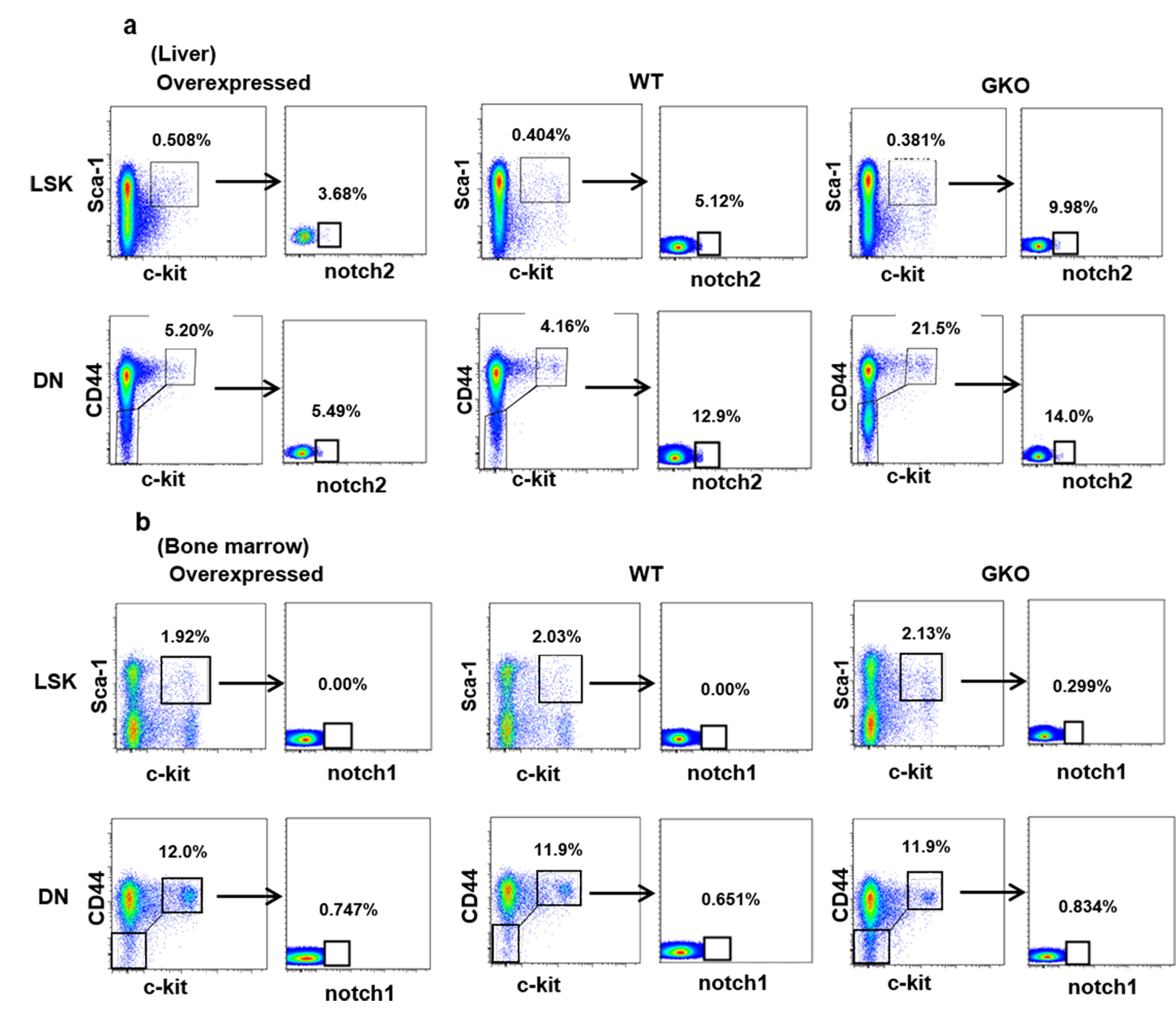
**

**Figure S3: The expression of notch1 or notch2 on the LSK and DN cells in the liver and BM of over-expressed IFN-γ mice, WT, and GKO mice.**

Flow cytometry plots showing the expression of notch2 on LSK cells and DN cells of C57BL/6j mice, GKO mice, and mouse livers overexpressing IFN-γ (a) and the expression of notch1 on LSK cells and DN cells of C57BL/6j mice, GKO mice, and mouse BM overexpressing IFN-γ (b) (n = 5−10 ). IFN-γ, interferon gamma; BM, bone marrow; LSK, Lineage^-^Sca-1^+^c-Kit^+^, SCA-1, Spinocerebellar Ataxia 1; DN, double negative; WT, wild-type; c-kit, KIT proto-oncogene, receptor tyrosine kinase; GKO, IFN-γ-deficient mice

**
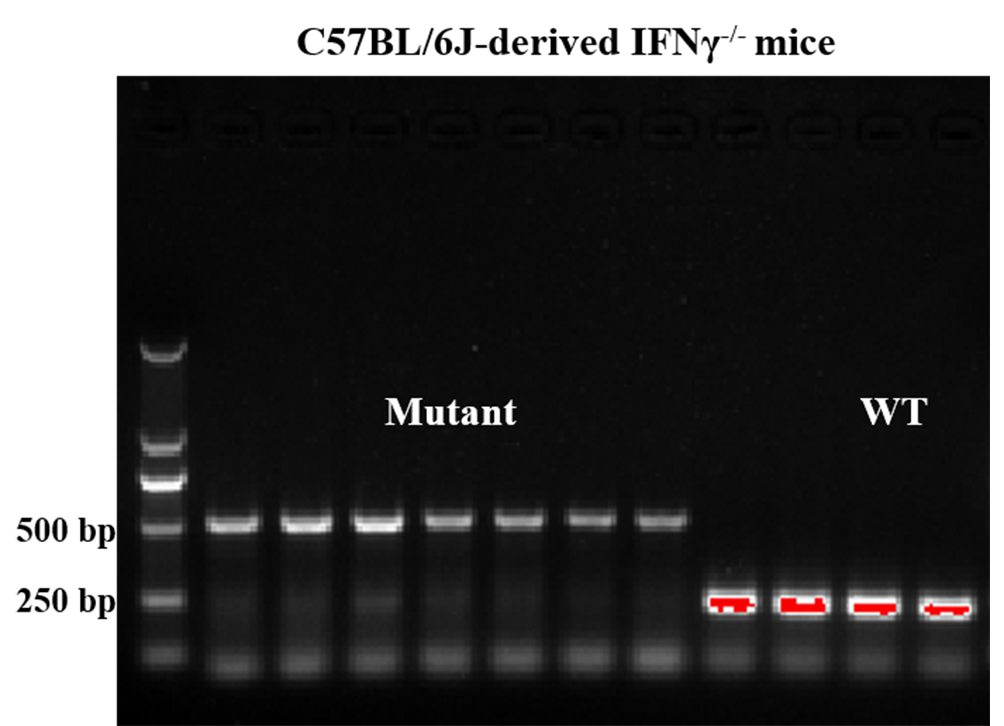
**

**Figure S4: Genotype identification of IFN-γ deficient mice.**

Claws of mice (to be identified mice and WT mice) were cut and placed in Eppendorf tubes with 250 μL proteinase K buffer and boiled for 10 min. After cooling to room temperature, 5μL of proteinase K was added, the tubes were inverted, and incubated overnight at 55 °C. Thereafter, the samples were briefly vortexed and boiled for 10 minutes. The samples were centrifuged at 1600 rcf at 4 °C and the supernatant was used as a template for PCR amplification. The amplified cDNA was then subjected to agarose gel electrophoresis. The band of WT mouse was 250 bp, and that of IFN-γ-deficient mice was 500 bp. IFN-γ, interferon gamma; WT, wild-type.
